# Persistence and treatment patterns of fixed combination drugs for glaucoma: a retrospective claims database study in Japan

**DOI:** 10.1186/s12886-020-01508-8

**Published:** 2020-06-10

**Authors:** Kenji Kashiwagi, Eriko Chono, Sarah Koesters, Poh Sin Yap

**Affiliations:** 1 Department of Ophthalmology, Faculty of Medicine, Yamanashi University, 1110 Shimokato, Chuo, Yamanashi Japan; 2 Novartis Pharma K.K, Tokyo, Japan; 3 Novartis Ireland Ltd., Dublin, Ireland; 4 Novartis Corporation Sdn. Bhd, Petaling Jaya, Malaysia

**Keywords:** Claims database, Combination therapy, Fixed combination, Glaucoma, Persistence, Topical treatment, Treatment pattern

## Abstract

**Background:**

Poor persistence with glaucoma therapy can lead to disease progression and subsequent blindness. Persistence with second-line glaucoma combination treatment in a Japanese real-world setting and whether it differed from fixed and unfixed combination drugs was investigated.

**Methods:**

This was a retrospective, non-interventional, cohort study using data from a Japanese medical claims database. Patients with glaucoma aged ≥20 years with a first drug claim for glaucoma treatment between 01 July 2005 and 30 October 2014 and with data for > 6 months before and after this first prescription were included. The primary endpoint was duration of drug persistence among glaucoma patients with and without the use of fixed combination drugs in the year following initiation of second-line combination treatment.

**Results:**

Of 1403 patients included in the analysis, 364 (25.94%) received fixed combination drugs and 1039 (74.06%) received unfixed combination drugs as second-line treatment. Baseline characteristics were generally comparable between the groups. A total of 39.01% of patients on fixed combination drugs, compared with 41.67% of patients on unfixed combination drugs, persisted on their glaucoma drugs 12 months post second-index date. Median persistence durations for the fixed combination drugs and unfixed combination drugs groups were 6 (95% confidence interval [CI]: 5–8) and 7 months (95% CI 6–9), respectively. Patients who received prostaglandin analogs (PGAs) were the most persistent with their treatment (*n* = 99, 12.84%). Patients diagnosed with primary open-angle glaucoma were less likely to experience treatment modification (hazard ratio [HR]: 0.800, 95% CI 0.649–0.986, *P* = 0.036), while those diagnosed with secondary glaucoma were more likely to experience treatment modification (HR: 1.678, 95% CI 1.231–2.288, *P* = 0.001) compared with glaucoma suspects.

**Conclusions:**

In this retrospective claims database study, the persistence rate of second-line glaucoma combination treatment was low, with no difference in persistence between glaucoma patients receiving unfixed combination drugs compared with fixed combination drugs. Patients on PGA showed greater persistence rates compared with other treatments.

## Background

Glaucoma, a life-long disease, is the leading cause of irreversible blindness in the world [1, 2]. This disease is characterized by a progressive neurodegeneration of the optic nerve and irreversible loss of retinal ganglion cells that results in visual field defects [3]. Increased intraocular pressure (IOP) is believed to be a major risk factor for glaucoma [4]. Clinical treatment aims to slow disease progression and maintain visual function by reducing IOP [5, 6].

The Japan Glaucoma Society recommends monotherapy with topical IOP-lowering agents as first-line treatment for open-angle glaucoma (OAG) and ocular hypertension (OHT) [7]. In cases where monotherapy is deemed insufficient, combination therapy with two or more IOP-lowering agents is recommended to achieve and maintain target IOP [8]. A fixed combination of prostaglandin analog (PGA) or carbonic anhydrase inhibitor (CAI) and a β-blocker (BB, timolol maleate 0.5%) is commonly used as second-line treatment [9, 10]. In Japan, fixed combination glaucoma drugs first became available for use in 2010. There are four different fixed combinations of PGAs and BBs (PGA/BB) and two of CAIs and BBs (CAI/BB) [7].

Adherence to and persistence with topical glaucoma treatment is low worldwide [2, 11, 12], which may potentially lead to disease progression and subsequent blindness [11, 13]. Both adherence and persistence are components of patient medication compliance [14]. Adherence is described as the degree to which the patient follows treatment instructions during a finite period of time, whereas the time until which a patient first discontinues use of medication is referred to as persistence [14]. Increasing the number of eye drop medications is associated with lower treatment adherence and patient persistence. Common barriers to persistence with multiple glaucoma medications include low self-efficacy, forgetfulness, difficulty with administration of eye drops, and complicated medication schedules [6, 15]. In addition, multiple medications may induce chronic ocular surface disease, such as superficial punctual keratitis because the preservatives in each ophthalmic solution may affect the cornea [16, 17]. Therefore, medications using fixed combinations of drugs are considered beneficial for glaucoma treatment as they improve patient medication adherence [18].

The objective of this real-world, retrospective claims database study in Japan was to investigate the persistence rate of second-line glaucoma combination treatment and compare persistence rates between fixed combination drugs and unfixed combination drugs. The real-world treatment pattern of patients with glaucoma in Japan is also described.

## Methods

### Study design

**Fig. 1:**
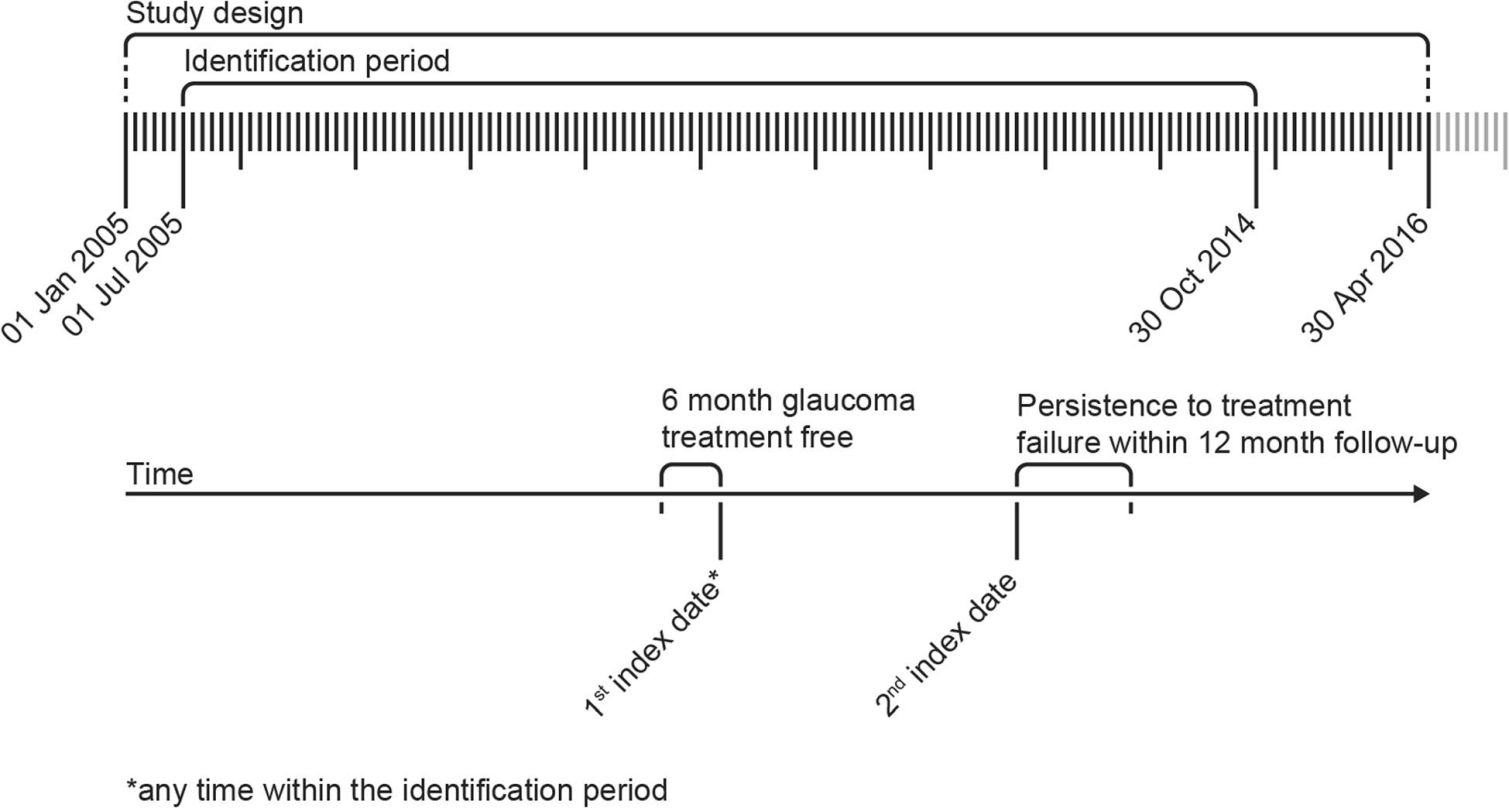
Study design

This was a retrospective, non-interventional, cohort study using data from the Japan Medical Claim Data Center (JMDC) claims database [19]. The study period began from 01 January 2005 and lasted until 30 April 2016 (Fig. 1).

The identification period started 6 months after the beginning of the study (01 July 2005) and ended 18 months before the end of the study period (30 October 2014).

The first index date was defined as the date of the first prescription of a glaucoma medication after diagnosis of glaucoma, with no such prescriptions in the past 6 months. The second index date was the date on which the second medication was added or a different medication was prescribed. Combination therapy was defined as the prescription of ≥2 classes of glaucoma medication, including one fixed combination drug prescription, within 30 days of the second index date. First- and second-line treatment was defined as the drug prescribed from first index date to second index date and from second-index date, respectively. The fixed combination drug group was defined as the group with at least one fixed combination drug prescribed.

The study was conducted in accordance with the Declaration of Helsinki and was approved by the Institutional Ethical Review Board of the Specified Nonprofit Organization clinical research promotion network Japan (CR-IRB-0052). As this study acquired secondary data from a medical claims database, written informed consent from patients was not required. The study investigators received only anonymized data from the JMDC; personal information of the patients was not disclosed.

### Data source

This study used the JMDC claims database which was provided by JMDC Inc. This database is a source of electronic and paper health insurance claims from over 50 health insurance associations in Japan [19]. Approximately 3,800,000 patient-level, anonymized data entries have been included in the JMDC since January 2005, accounting for 3.0% of the entire Japanese population. Patients’ data were securely anonymized by JMDC using Information MediC4, “irreversible anonymous aggregation technology” that heavily encrypted the information such that individuals could not be identified [19].

This database includes information on: (i) age, (ii) gender, (iii) dates of healthcare coverage, (iv) healthcare claims (treatments, procedures, laboratory and diagnostic tests performed, healthcare visits, and treatment details, such as Anatomical Therapeutic Chemical codes, product/brand, daily dosage, duration), (v) disease diagnoses (Standard Disease Codes compatible with the International Classification of Diseases, 10th Revision [ICD-10] codes [20], and (vi) costs associated with healthcare claims.

The following components of the database were used in this retrospective analysis: (i) age at index date (in years), (ii) gender, (iii) year of index date(s), (iv) date(s) of prescription, (v) drug category, (vi) smoking status, (vii) medical history, (viii) type of medication, (ix) type of glaucoma, (x) hospital size, and (xi) blood pressure. Glaucoma treatment was categorized as unfixed combinations of any of PGA, BB, CAI, α-blocker, α-agonist, and other (rho kinase inhibitor, osmotic diuretic, or autonomic agent) or as fixed combinations (specifically either PGA/BB or CAI/BB). Follow-up time and persistence were recorded from the second index date. Patient demographic characteristics were measured at the second index date.

### Patients

Patients aged ≥20 years, diagnosed with glaucoma (primary OAG, glaucoma secondary to eye trauma, glaucoma secondary to eye inflammation, glaucoma secondary to other eye disorders, glaucoma secondary to drugs, or other or unspecified glaucoma) between 1 January 2005 and 30 October 2014, with a first drug claim for glaucoma treatment between 1 July 2005 and 30 October 2014 (first index date), with data ≥6 months before and ≥ 6 months after the first index date, were included. Patients who received second-line combination treatment and for whom data for 12 months after the second index date were available were also included in the analysis.

### Study endpoints

The primary endpoint was duration of persistence, in months, in the year following initiation of second-line combination treatment, comparing this between patients who received fixed combination treatment with unfixed combination treatment. In Japan, first-line treatment for glaucoma is commonly monotherapy; to evaluate the persistence of combination therapy, persistence was defined as the duration from the initiation of second-line combination treatment until treatment modification. Treatment modification was defined as any one of the following: (i) changing to a new medication indicated for glaucoma after starting the second-line combination treatment, (ii) medication reduction after starting the second-line combination treatment, lasting for 6 months or more, and (iii) first claim for laser treatment or surgery for glaucoma after starting the second-line combination treatment. Exploratory analyses included the association between treatment modification and patient demographics and clinical characteristics, as well as stratification by fixed combination treatment.

The secondary endpoints included the following: 1) frequency and proportion of the first- and second-line glaucoma treatment by drug category from 2005 to 2014, 2) duration of first-line treatment before the switch to second-line treatment by category, 3) the frequency and proportion of patients who switched to second-line treatment, including those who switched to or added on another drug in the third-line, stopped combination therapy, or stayed on combination therapy, and 4) duration of second-line treatment among those who went on to receive third-line treatment, by category.

### Statistics

#### Sample size

A total of 0.2% of the 3,800,000 patients, (*n* = 7600) in JMDC were expected to meet the inclusion criteria. Of these patients, assuming around 40% of patients were using combination drugs (either fixed or unfixed) and patients were equally distributed between the two groups, ~ 1520 patients were expected to receive either fixed or unfixed combination drugs. A persistence rate of ~ 30% within a year after starting the second-line combination treatment for patients on fixed combination drug and ~ 25% for patients on an unfixed combination was estimated based on the study by Higginbotham [21]. Using a binomial distribution, the 95% confidence interval (CI) for persistence in patients with fixed and unfixed combination drugs was 0.3 ± 0.023 and 0.25 ± 0.022, respectively, and these precisions were expected to be sufficient to interpret the study results.

#### Statistical methods

Kaplan-Meïer curves were used to investigate the crude association of persistence among patients with fixed and unfixed combination drugs. A log-rank test was used to test the association.

A multivariable Cox proportional hazards model was fitted to estimate the effect of fixed and unfixed medication groups on the hazard of treatment modification, adjusting for potential confounders of age, gender, first-line treatment, smoking status, type of glaucoma, duration of first-line treatment, blood pressure, and hospital size. All estimated effects were reported with a 95% CI, regardless of their significance. The estimated adjusted hazard ratio (HR) between the two treatment groups and their associated 95% CI and *P* value were reported.

Descriptive statistics were performed to describe the treatment patterns. Statistical analyses were performed using SAS Studio 9.4 (SAS Institute, Cary, NC, USA). Missing values were excluded for this analysis and a two-tailed *P* value of < 0.05 was considered statistically significant.

## Results

### Patient disposition and baseline characteristics

**Fig. 2:**
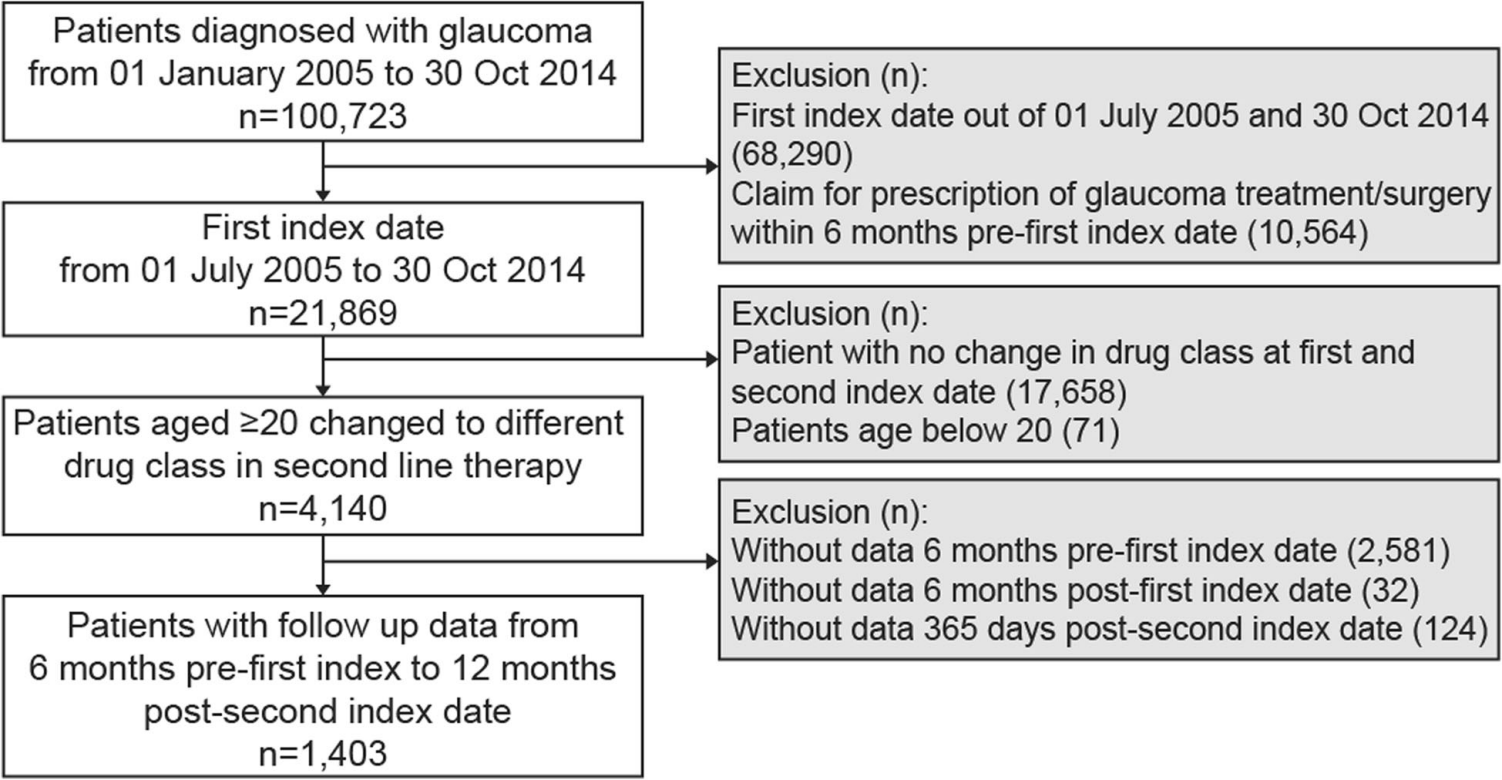
Patient disposition

Of the 100,723 glaucoma patients identified from the JMDC, 1403 met the eligibility criteria and were included in the analysis (Fig. 2). Of these 1403 patients, 363 (25.87%) were glaucoma suspects, 288 (20.53%) had primary OAG, 7 (0.50%) had glaucoma secondary to eye inflammation, 48 (3.42%) had glaucoma secondary to eye disorders, 7 (0.50%) had glaucoma secondary to drugs, and 690 (49.18%) had unspecified glaucoma (Table 1). For first-line treatment, 83 (5.92%) patients received fixed combination drugs and 1320 (94.08%) received unfixed combination drugs. For second-line treatment, 364 (25.94%) patients received fixed combination and 1039 (74.06%) received unfixed combination drugs.

**Table 1:** Baseline characteristics

Characteristic	Fixed combination *n* = 364	Unfixed combination *n* = 1039	Overall *N* = 1403
Age, mean (SD)	49.62 (10.72)	49.76 (10.03)	49.72 (10.21)
Age, n (%)			
20–34 years	26 (7.14)	72 (6.93)	98 (6.99)
35–49 years	169 (46.43)	454 (43.70)	623 (44.40)
50–64 years	137 (37.64)	448 (43.12)	585 (41.70)
≥ 65 years	32 (8.79)	65 (6.26)	97 (6.91)
Gender, n (%)			
Male	215 (59.07)	594 (57.17)	809 (57.66)
Female	149 (40.93)	445 (42.83)	594 (42.34)
Systolic blood pressure, mmHg, mean (SD)	122.4 (15.77) *n* = 251	124.76 (17.06) *n* = 788	124.19 (16.78) *n* = 1039
Diastolic blood pressure, mmHg, mean (SD)	76.28 (11.57)	76.72 (11.58)	76.62 (11.57)
Smoking status, *n* (%)			
Non-smoker	188 (51.65)	555 (53.42)	743 (52.96)
Current smoker	53 (14.56)	140 (13.47)	193 (13.76)
Missing/unknown	123 (33.79)	344 (33.11)	467 (33.29)
Type of glaucoma (ICD-10 code), n (%)			
Glaucoma suspect (H40.0)	85 (23.35)	278 (26.76)	363 (25.87)
Primary open-angle glaucoma (H40.1)	57 (15.66)	231 (22.23)	288 (20.53)
Glaucoma secondary to eye inflammation (H40.4)	3 (0.82)	4 (0.38)	7 (0.5)
Glaucoma secondary to other eye disorders (H40.5)	21 (5.77)	27 (2.60)	48 (3.42)
Glaucoma secondary to drugs (H40.6)	1 (0.27)	6 (0.58)	7 (0.5)
Unspecified glaucoma (H40.9)	197 (54.12)	493 (47.45)	690 (49.18)
Hospital size, *n* (%)			
≤ 19 hospital beds	235 (64.56)	741 (71.32)	976 (69.57)
20–99 hospital beds	9 (2.47)	23 (2.21)	32 (2.28)
100–199 hospital beds	8 (2.2)	21 (2.02)	29 (2.07)
200–299 hospital beds	10 (2.75)	25 (2.41)	35 (2.49)
300–499 hospital beds	22 (6.04)	54 (5.20)	76 (5.42)
≥ 500 hospital beds	80 (21.98)	175 (16.84)	255 (18.18)
Duration of second-line treatment (months), mean (SD)	13.4 (17.05)	12.25 (14.90)	12.55 (15.49)

*ICD* International Classification of Diseases, *N* total number of patients, *n* number of patients, *SD* standard deviation

Baseline characteristics were generally comparable between the fixed and unfixed combination drug groups (Table 1). The mean (standard deviation [SD]) age of patients in the fixed and unfixed combination drug groups was 49.62 (10.72) and 49.76 (10.03) years, respectively. The majority of patients were male in both groups.

### Duration of persistence with second-line treatment

**Fig. 3:**
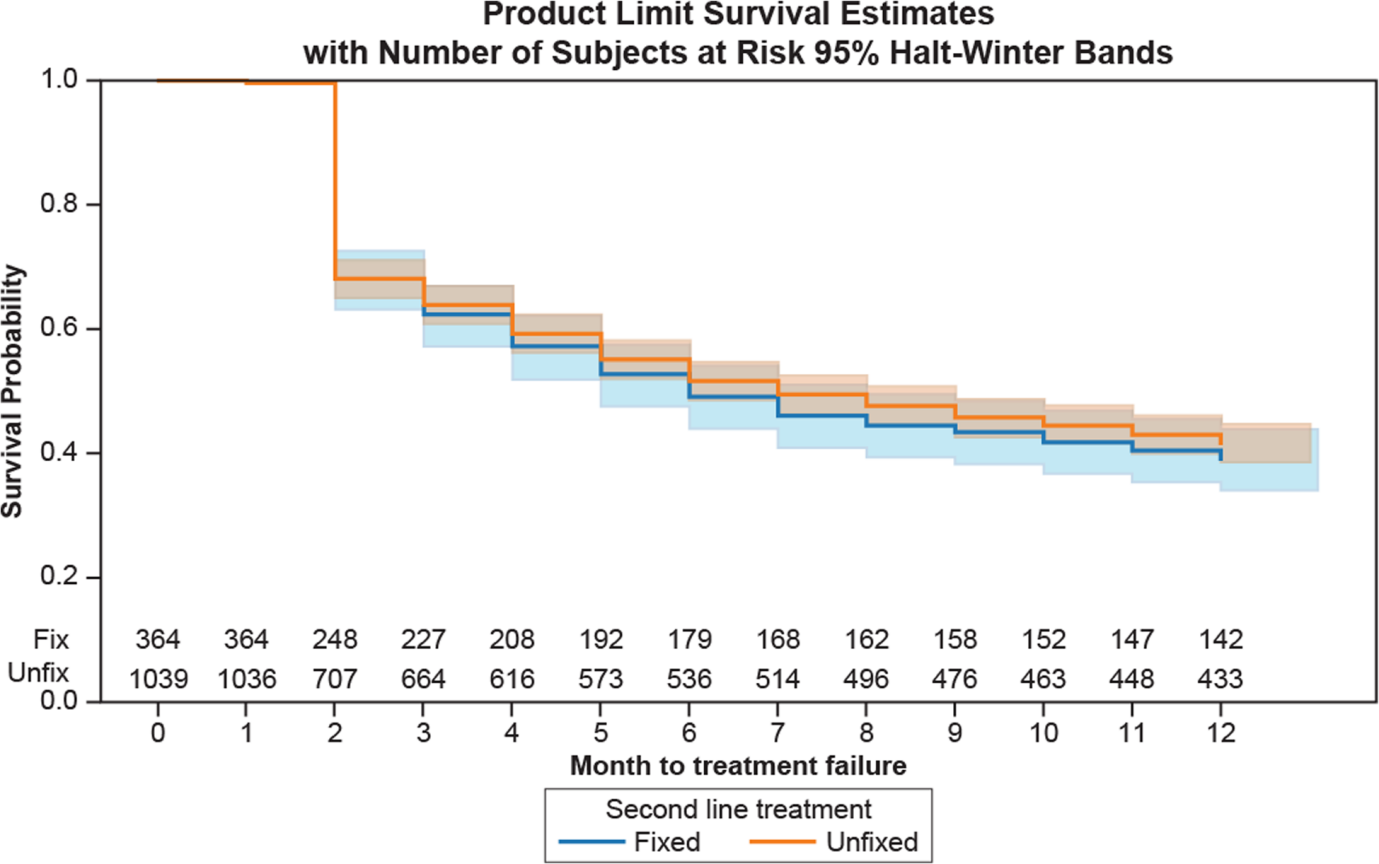
Kaplan-Meïer plot for treatment modification-free time with log-rank. Fixed, fixed combination drug group; Unfixed, unfixed combination drug group

A total of 39.01% of patients on fixed combination drugs stayed on their glaucoma drugs 12 months after starting the second-line combination treatment compared with 41.67% of patients on unfixed combination drugs (Fig. 3). The median persistence time for the fixed and unfixed drug combination groups was 6 months (95% CI 5–8) and 7 months (95% CI 6–9), respectively. Patients who received PGAs were the most persistent with their treatment (99 patients, 12.84%; data not shown). From Months 3 to 12 the survival probability of the fixed combination drug group was slightly lower than that of the unfixed combination drug group (Fig. 3).

### Factors associated with second-line treatment modification

Based on the unadjusted HR model, when compared with glaucoma suspects, patients diagnosed with primary OAG were less likely to experience treatment modification (HR: 0.800, 95% CI 0.649–0.986, *P* = 0.036), while those diagnosed with secondary glaucoma were more likely to experience treatment modification (HR: 1.678, 95% CI 1.231–2.288, *P* = 0.001, Table 2). There was a decreased risk of treatment modification for every 1 month increase in the duration of first-line treatment (HR: 0.987, 95% CI 0.982–0.993, *P* < 0.001, Table 2). When the model was adjusted for confounding factors similar findings were observed in the subgroup of patients with secondary glaucoma (Table 2). Other baseline characteristics tested did not affect treatment modification in the unadjusted and adjusted models (Table 2).

**Table 2:** Unadjusted and adjusted HR for treatment modification

Variable	Unadjusted HR (95% CI)	*P* value	Adjusted HR (95% CI)	*P* value
Combination drug				
With fixed combination drug	1.065 (0.913, 1.243)	0.4205	0.988 (0.811, 1.203)	0.9016
Without fixed combination drug	Referent		Referent	
Age (years)	1.000 (0.994, 1.007)	0.9078	0.993 (0.984, 1.003)	0.1634
Gender				
Male	Referent		Referent	
Female	1.025 (0.893, 1.177)	0.722	1.048 (0.863, 1.273)	0.6338
Smoking status				
Non-smoker	Referent		Referent	
Current smoker	1.221 (0.996, 1.496)	0.0546	1.216 (0.982, 1.506)	0.0729
Type of glaucoma (ICD-10 code)				
Glaucoma suspect (H40.0)	Referent		Referent	
Primary open angle glaucoma (H40.1)	0.800 (0.649, 0.986)	0.0361	0.834 (0.643, 1.082)	0.1719
Secondary glaucoma (H40.3 to H40.6)	1.678 (1.231, 2.288)	0.0011	1.512 (1.003, 2.280)	0.0484
Unspecified glaucoma (H40.9)	1.006 (0.853, 1.187)	0.9403	1.070 (0.868, 1.319)	0.5243
Hospital size				
≤ 19 hospital beds	Referent		Referent	
20–499 hospital beds	0.912 (0.734, 1.135)	0.4098	0.879 (0.667, 1.158)	0.3582
≥ 500 hospital beds	0.852 (0.717, 1.013)	0.0702	0.859 (0.687, 1.074)	0.1832
Systolic blood pressure (mmHg)	1.005 (1, 1.009)	0.0618	1.002 (0.994, 1.011)	0.5637
Diastolic blood pressure (mmHg)	1.007 (1, 1.014)	0.0595	1.005 (0.993, 1.017)	0.4169
Duration of first-line treatment (months)	0.987 (0.982, 0.993)	< 0.0001	0.988 (0.981, 0.994)	0.0002

*CI* confidence interval, *HR* hazard ratio, *ICD* International Classification of Diseases

The majority of patients on first-line treatment received a single category of drug, for both unfixed and fixed combination therapy (*n* = 1218 [92.27%] and *n* = 63 [75.90%], respectively) (Table 3). Most patients on second-line treatment were administered two different categories of glaucoma drugs, in both the unfixed and fixed combination therapy groups (*n* = 812 [78.15%] and *n* = 306 [84.07%], respectively, Table 3).

**Table 3:** Number of drug categories for first-line and second-line treatment

		Number of drug categories				
		1	2	3	4	5
First-line treatment, *n* (%)	Unfixed combination (*n* = 1320)	1218 (92.27)	89 (6.74)	11 (0.83)	2 (0.15)	–
	Fixed combination (*n* = 83)	63 (75.90)	18 (21.69)	2 (2.41)	–	–
	Total (N = 1403)	1281 (91.30)	107 (7.63)	13 (0.93)	2 (0.14)	–
Second-line treatment, n (%)	Unfixed combination (n = 364)	136 (13.09)	812 (78.15)	84 (8.08)	6 (0.58)	1 (0.10)
	Fixed combination (n = 1039)	4 (1.10)	306 (84.07)	47 (12.91)	6 (1.65)	1 (0.27)
	Total (N = 1403)	140 (9.98)	1118 (79.69)	131 (9.34)	12 (0.86)	2 (0.14)

*N* total number of patients, *n* number of patients

When stratified by drug name among patients using fixed combination drugs, those who received a fixed combination of travoprost/timolol maleate were less likely to experience treatment modification than patients receiving any other combination (HR: 0.670, 95% CI 0.477–0.941, *P* = 0.0208) in the unadjusted model (Table 4). A similar effect was observed when the analysis was adjusted for confounding factors (HR: 0.578, 95% CI 0.377–0.886, *P* = 0.0119).

**Table 4:** Unadjusted and adjusted HR for treatment modification stratified by drug category

Second-line combination drug	Unadjusted HR (95% CI)	*P* value	Adjusted HR (95% CI)	*P* value
Fixed combination				
PGA/BB				
Latanoprost/ Timolol maleate	1.377 (0.959 1.977)	0.0832	1.322 (0.83 2.106)	0.2397
Travoprost/ Timolol maleate	0.670 (0.477 0.941)	0.0208	0.578 (0.377 0.886)	0.0119
CAI/BB				
Brinzolamide/ Timolol maleate	0.852 (0.499 1.453)	0.5561	0.802 (0.394 1.631)	0.5418
Dorzolamide hydrochloride/ Timolol maleate	0.998 (0.751 1.326)	0.9867	0.862 (0.6 1.238)	0.4211
Unfixed combination				
PGA	0.527 (0.404 0.687)	< 0.0001	0.549 (0.39 0.771)	0.0006
CAI	3.077 (1.721 5.502)	0.0002	3.603 (1.77 7.334)	0.0004
BB	1.867 (1.388 2.513)	< 0.0001	2.014 (1.386 2.926)	0.0002
Others^a^	Reference		Reference	
PGA and BB	0.717 (0.553 0.929)	0.0118	0.788 (0.57 1.091)	0.1508
PGA and CAI	0.704 (0.485 1.021)	0.0642	0.81 (0.513 1.28)	0.3678
CAI and BB	1.181 (0.806 1.731)	0.3936	1.082 (0.671 1.744)	0.7461
PGA and BB and CAI	0.846 (0.496 1.444)	0.5406	0.871 (0.441 1.722)	0.692

^a^Others included the following: α-blocker, α-agonist, rho kinase inhibitor, osmotic diuretic and autonomic agent BB, β blocker; CAI, carbonic anhydrase inhibitor; CI, confidence interval; HR, hazard ratio; PGA, prostaglandin analog

Patients receiving PGA monotherapy (HR: 0.527, 95% CI 0.404–0.687, *P* < 0.0001) and PGA plus BB unfixed combination therapy (HR: 0.717, 95% CI 0.553–0.929, *P* = 0.0118) were less likely to experience treatment modification when compared with patients who received other treatments (Table 4). In contrast, patients who received CAI (HR: 3.077, 95% CI 1.721–5.502, *P* = 0.0002) or BB (HR: 1.867, 95% CI 1.388–2.513, *P* < 0.0001) monotherapy were more likely to have treatment modification when compared with patients who received other treatments (Table 4). Again, similar patterns were observed when the model was adjusted for patients who received PGA (HR: 0.549, 95% CI 0.390–0.771, *P* = 0.0006), CAI (HR: 3.603, 95% CI 1.770–7.334, *P* = 0.0004), or BB monotherapy (HR: 2.014, 95% CI 1.386–2.926, *P* = 0.0002) (Table 4).

### Treatment patterns

**Fig. 4:**
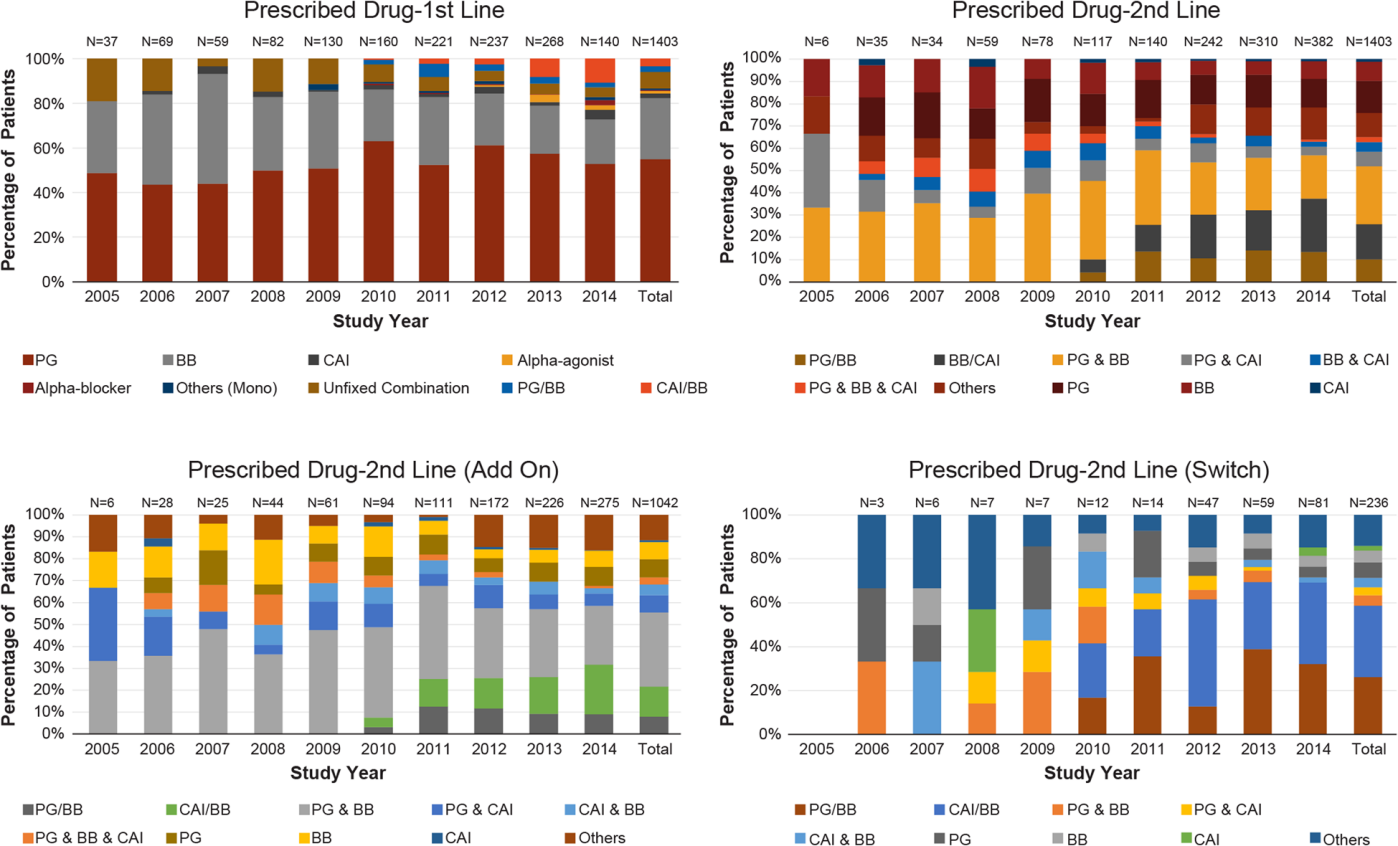
Treatment pattern for glaucoma patients; a) first-line, b) second-line c) drugs added at second-line, d) switch at second-line. BB, β blocker; CAI, carbonic anhydrase inhibitor; CAI/BB, treatment with CAI and BB fixed combination drug; n number of patients in group, N total number of patients; Others, other combination therapy, Others (Mono), monotherapy with rho kinase inhibitor, osmotic diuretic or autonomic agent; PGA, prostaglandin analog; PGA/BB, treatment with PGA and BB fixed combination drug; Unfixed combination, combination therapy with separate drugs

In the period from January 2005 to October 2014, of the 1403 patients who received first-line treatment, PGAs were the most commonly used drugs (*n* = 771, 54.95%) followed by BBs (*n* = 386, 27.51%, Fig. 4a). Among the fixed combination drugs, CAI/BB was most commonly used as first-line treatment (*n* = 49, 3.49%, Fig. 4a). Overall, BB was the most commonly added on drug for patients on first-line treatment (*n* = 325, 23.16%) followed by PGA (*n* = 256, 18.24%) and CAI (*n* = 173, 12.33%).

Of the 1403 patients who received second-line treatments, 1039 patients (74.06%) were on unfixed combination treatment while 364 patients (25.94%) were on fixed combination treatment (Table 3). Of those, 125 patients persisted on their first-line treatment, 1042 patients added on drugs (Fig. 4c) while 236 patients switched drugs (Fig. 4d). Of the 125 patients who persisted with their first-line treatment, patients receiving PGA were the most persistent (*n* = 99, 12.84%) followed by BB (n = 25, 6.48%) and CAI (n = 1, 3.45%, data not shown). The most common second-line treatment was the unfixed combination of PGA plus BB (*n* = 365, 26.02%) followed by the fixed combination CAI/BB (*n* = 219, 15.61%, Fig. 4b). Of the 236 patients who switched during second-line treatment, the most commonly switched treatment was CAI/BB, followed by PGA/BB (Fig. 4d).

### Treatment duration

Patients who received fixed combination second-line treatment had a shorter mean duration (±SD) of their first-line treatment before they switched drugs (8.19 ± 8.98 months) whereas patients who received an unfixed combination second-line treatment had a longer duration of their first-line treatment before switching (16.94 ± 18.05 months, Table 5). Of the patients on second-line switched from first-line treatment, most patients stayed on combination therapy (37.50 and 33.33%, respectively, Table 5). Patients who received an α-agonist had the shortest duration of first-line treatment (4.36 ± 6.54 months, Table 5).

**Table 5:** Treatment duration by drug

Treatment Pattern	
Patients switched from first-line treatment, *n*	182
Duration of the first-line treatment, months (mean ± SD)	
Fixed combination	8.19 ± 8.98
Unfixed combination	16.94 ± 18.05
Duration of first-line treatment, months (mean ± SD)	
PGA	12.63 ± 14.88
BB	13.9 ± 16.79
CAI	5.14 ± 7.20
α-blocker	6.71 ± 5.91
α-agonist	4.36 ± 6.54
Others	17 ± 22.28
PGA/BB	8.47 ± 9.39
CAI/BB	5.2 ± 6.83
Of those switched to fixed combination as second-line, n (%)	*n* = 104
Switched to another drug in the third-line	22 (21.15)
Added on another drug in the third-line	18 (17.31)
Dropped the combination therapy	25 (24.04)
Stayed on the combination therapy	39 (37.50)
Of those switched to unfixed combination as second-line, n (%)	n = 78
Switched to another drug in the third-line	16 (20.51)
Added on another drug in the third-line	15 (19.23)
Dropped the combination therapy	21 (26.92)
Stayed on the combination therapy	26 (33.33)
Duration of second-line treatment (added on in the third-line), months (mean ± SD)	
PGA	8.08 ± 4.37
BB	7.02 ± 4.55
CAI	6.52 ± 4.94
α-blocker	7.00 ± 5.77
α-agonist	5.58 ± 4.60
Others	4.86 ± 3.63
PGA/BB	6.90 ± 4.61
CAI/BB	7.03 ± 4.46
Duration of second-line treatment (switched in the third-line), month (mean ± SD)	
PGA	7.08 ± 4.54
BB	7.39 ± 4.38
CAI	4.40 ± 2.88
α-blocker	10.00 ± 2.00
α-agonist	7.00 ± 7.07
Others^a^	4.75 ± 4.86
PGA/BB	7.14 ± 4.64
CAI/BB	2.92 ± 2.78

*BB* β blocker, *CAI* carbonic anhydrase inhibitor, *CAI/BB* CAI and BB fixed combination drug, N, total number of patients, *n* number of patients, *PGA* prostaglandin analog, *PGA/BB* PGA and BB fixed combination drug, *SD* standard deviation

^a^Others included: rho kinase inhibitor, osmotic diuretic and autonomic agent

Patients who received a PGA as second-line treatment remained on their treatment for the longest duration before the addition of drugs for third-line treatment (8.08 ± 4.37 months), while patients who received other types of second-line treatment remained on their treatment for the shortest time period before adding on drugs for third-line treatment (4.86 ± 3.63 months, Table 5). Patients who received an α-blocker as second-line treatment remained on their treatment for the longest duration before switching agents as third-line treatment (10.00 ± 2.00 months), while patients who received CAI/BB remained on their treatment for the shortest duration before switching agents as third-line treatment (2.92 ± 2.78 months, Table 5).

## Discussion

To the best of our knowledge, this is the first study of persistence until treatment modification with second-line glaucoma combination treatment. In Japan, fixed combination glaucoma drugs are often used as second-line treatment and the persistence between fixed drug combinations and unfixed drug combinations was compared. Patients with glaucoma receiving second-line treatment with an unfixed drug combination showed slightly better persistence than recipients of a fixed combination of drugs, although the difference was not statistically significant. Patients were stratified by fixed combination to explore whether persistence varied by different fixed combinations. Patients who received travoprost/timolol maleate fixed combination, PGA monotherapy, or the combination of PGA plus BB (unfixed) were less likely to have treatment modification compared with those who received other glaucoma treatments. Patients who received CAI or BB monotherapy were more likely to have treatment modification than those receiving other treatments. This study of treatment patterns revealed PGA to be the most commonly used first-line monotherapy, CAI/BB as the most common second-line fixed combination treatment, and BB plus PGA as the most common second-line unfixed combination therapy. These results are consistent with past reports [22–27].

Persistence is generally low in glaucoma patients, which could potentially lead to disease progression and subsequent blindness [11, 13]. An increase in the number of eye drop medications is associated with lower patient persistence; difficulty with drop administration, medication schedules, and forgetfulness are also considered to be the other main reasons for low persistence. In this study patients with glaucoma who were on an unfixed combination as second-line treatment showed slightly better persistence compared with the fixed combination drug group; however the difference was not statistically significant. This is in agreement with an earlier study conducted in an Asian population [13] but in contrast with earlier studies conducted in Caucasians [2, 21, 28] which reported lower persistence with increasing numbers of bottles of topical drug used. Patients’ attitude towards glaucoma treatment varies by region and country, which may explain the observed difference in patient persistency between the current study and studies in Caucasians. Of the patients who were on second-line combination treatment, 84.07% of patients in the fixed combination therapy group and 78.15% of patients in the unfixed combination therapy group received two different categories of drug. This implies that patients in the fixed combination therapy group were treated with three different classes of glaucoma drug, while those in the unfixed combination therapy group were treated with two, which may affect persistence with treatment. There is also the possibility that patients with more advanced disease progression were prescribed fixed combination drugs compared with unfixed combination drugs, which may explain the difference in persistence rate between these groups.

This study uncovered the risk factors associated with treatment modification, which has not been previously reported. Longer duration of first-line treatment posed a significantly lower risk factor for eventual treatment modification (HR: 0.987, 95%CI 0.982–0.993, *P* < 0.001, Table 2). This observation could be explained by patients with a shorter duration of first-line treatment being generally (and expectedly) in a more advanced disease state and therefore more likely to experience treatment resistance compared with others. Patients with secondary glaucoma showed a tendency towards higher risk of treatment modification than glaucoma suspects. The former group of patients were more likely to be subjected to treatment for their underlying condition rather than remaining on existing glaucoma therapy [7, 29].

The treatment patterns in Japan reported in this study are consistent with earlier studies conducted in various countries. The most commonly used first-line monotherapy was a PGA [22, 23, 25, 26], while CAI/BB was the most commonly used fixed combination as first- and second-line treatment [24, 27], and the most commonly used second-line unfixed combination was PGA plus BB. Drug-persistence was highest in patients who received a PGA [24, 30].

The limitations of this study are those inherent to all studies using administrative claims data [31]. Data collected in the JMDC claims database are based on a convenience sample. It has less coverage of the elderly population, those of 65–74 years of age (inclusive), and no coverage of persons aged 75 years or older. As a result, it is not a random sample of the Japanese population. This limits the extent to which the results from this study may be applied to other populations. Administrative claims data were recorded for transactions of reimbursement for healthcare and are not specifically designed for outcomes research purposes. Administrative claims data did not collect clinical information e.g. average IOP or visual field data, which limit the evaluation of the association of disease severity and persistence. This analysis may overestimate pharmacy claims for a filled prescription for actual drug exposure. These data are subject to coding errors and data omissions; however, independent, double-programming ensured optimal quality of the analysis.

In conclusion, persistence with second-line glaucoma combination treatment is low and there is no difference in the persistence observed in glaucoma patients who received unfixed combination treatment compared with patients who received a fixed drug combination. Further study is required to identify persistence rates in patients with glaucoma at different stages of disease progression. The results from this real-world study describing the treatment patterns in glaucoma may be useful for ophthalmologists in management of this disease.

## Data Availability

The data that support the findings of this study are available from JMDC Inc. but restrictions apply to the availability of these data, which were used under license for the current study, and so are not publicly available. Data are however available from the authors upon reasonable request and with permission of JMDC Inc.

## Abbreviations

BB: β-blocker
CAI: carbonic anhydrase inhibitor
CI: confidence interval
HR: hazard ratio
ICD-10: International Classification of Diseases, 10th Revision
IOP: intraocular pressure
JMDC: Japan Medical Claim Data Center
OAG: open-angle glaucoma
OHT: ocular hypertension
PGA: prostaglandin analog
SD: standard deviation
